# Description of *Crassolabium persicum* sp. n. (Nematoda, Dorylaimida, Qudsianematidae), an interesting species from Iran

**DOI:** 10.3897/zookeys.203.3248

**Published:** 2012-06-20

**Authors:** Habibeh Jabbari, Gholamreza Niknam, Maria Teresa Vinciguerra, Shalaleh Moslehi, Joaquín Abolafia, Reyes Peña-Santiago

**Affiliations:** 1 Nematology Lab., Faculty of Agriculture, University of Tabriz, Tabriz, Iran; 2 Dipartimento di Biologia Animale, Universita di Catania, via Androne 81, I–95124 Catania, Italy; 3 Departamento de Biología Animal, Biología Vegetal y Ecología, Universidad de Jaén, Campus ‘Las Lagunillas’ s/n, Edificio B3, 23071 Jaén, Spain

**Keywords:** *Crassolabium*, description, Dorylaimida, Iran, morphology, nematodes, new species, taxonomy

## Abstract

A new species of the genus *Crassolabium*, *Crassolabium persicum* **sp. n.**, collected from Arasbaran rangelands of Iran, is described and illustrated. It is characterized by its body 1.92–2.40 mm long, lip region offset by constriction and 17–19 μm wide, odontostyle 16–19 μm long with aperture occupying less than one–third (27–30%) its length, neck 428–690 μm long, pharyngeal expansion 369–390 μm long or occupying 54–56% of total neck length, female genital system amphidelphic, uterus bipartite and 162–218 μm long or 2.3–3.5 times as long as body diameter, *pars refringens vaginae* well developed, *V* = 54–57.5, vulva longitudinal, prerectum bearing a blind sac, tail conical with rounded tip to conoid (25–36 μm, *c*=60–69, *c’*=0.5–0.9), spicules 68–72 μm long, precloacal pair of supplements far (22–27 μm) from cloacal aperture, and 13–17 shortly spaced ventromedian supplements with hiatus. The new taxon is compared in depth to its relatives in *Crassolabium* as well as other similar species of *Aporcelaimellus* and *Amblydorylaimus*.

## Introduction

The genus *Crassolabium* Yeates, 1967 (syn. *Thonus* Thorne, 1974) is a highly diverse and worldwide distributed dorylaimid taxon. According to the recent compendium provided by Peña-Santiago and Ciobanu (2011), it includes 33 valid species known to occur in 47 countries, islands and archipelagos. It is a rather heterogeneous nematode group, whose differentiation from the genera *Aporcelaimellus* Heyns, 1965 (syn. *Takamangai* Yeates, 1967) and *Labronema* Thorne, 1939 is occasionally intricate.

The dorylaimid fauna of Iran has not traditionally received much attention. This situation has significantly changed during recent years, when a series of contributions – see, for instance, Pedram et al. (2011) and the references cited therein – have revealed it is rich in species, many of them previously unknown. Concerning presence of the genus *Crassolabium*, in Iran, Mowlavi et al. (in press) very recently reported the presence of *Crassolabium rhopalocercum* (de Man, 1876) Peña-Santiago and Ciobanu 2008, but there is no other available information.

During a general nematological survey in natural and cultivated areas, a few female and male specimens belonging to this genus were collected. Its study revealed this material belongs to a non-described and interesting species, which is the matter of this contribution.

## Material and methods

Soil samples were collected from Mahmood Abad, Arasbaran rangelands, northwest Iran, during 2009–2010. The nematodes were extracted by a modified combined sieving and centrifugation flotation method (Jenkins 1964), processed to anhydrous glycerine following De Grisse’s (1969) technique, and mounted on permanent glass slides for handling. Morphometric data were obtained using a drawing tube attached to an Olympus BX 41 light microscope. Morphometrics included de Man’s indices and other measurements traditionally diagnostic for the group. Some of the best-preserved specimens were photographed with a Nikon Eclipse 80i microscope and a Nikon DS digital camera. Raw photographs were edited using Adobe® Photoshop® CS. Drawings were made using a *camera lucida* attached to Nikon Eclipse 80i microscope.

## Taxonomy

### Crassolabium persicum sp. n.

urn:lsid:zoobank.org:act:E8DCBD3A-1F12-4390-98C4-F2AFCA0A1CF9

http://species-id.net/wiki/Crassolabium_persicum

Figures 1 2

#### Material examined.

Five females and four males, in excellent state of preservation.

#### Measurements.

See Table 1.

**Table 1.** Morphometric data of *Crassolabium persicum* sp. n. Measurements in μm except L in mm, and in the form: mean ± standard deviation (range).

	**Holotype**	**Paratypes**	**Paratypes**
**Character n**	♀	**5**♀♀	**4**♂♂
L	2.33	2.22±0.19 (1.92–2.40)	2.24±0.09 (2.13–2.34)
a	34	31.9±1.4 (30–34)	34.2±2.4 (32–38)
b	3.5	4.0±0.36 (3.5–4.2)	4.0±0.4 (3.7–4.7)
c	68	64.8±3.5 (60–69)	69.5±3.1 (63–72)
c’	0.7	0.74±0.14 (0.5–0.9)	0.75±0.09 (0.6–0.8)
V	57	57±1.75 (54–57.5)	–
Lip region diameter	17.5	17.8±0.6 (17–19)	18.2±2.5 (17–19)
Odontostyle length	19	17.5±0.9 (16–19)	18.6±1.2 (18–20)
Odontophore length	27.5	28.5±1.4 (26–31)	28.6±1.9 (27–31)
Guiding ring – anterior end	10	11.0±1.3 (10–12)	11.2±0.5 (11–12)
Neck length	690	428–690	542–569
Pharyngeal expansion length	380	369–390	308–315
Body diameter at neck base	65	68±2.1 (58–71)	62.6±7.6 (57–64)
mid–body	69	69.5±3.5 (63–74)	65.5±4.0 (61–69)
anus/cloaca	43	42.2±2.0 (38–44)	40.1±1.8 (39–43)
Rectum length	34	35.1±6.5 (29–40)	35.0±0.9 (34–36)
Prerectum length	68	67.5±11.5 (57–102)	70±9.0 (62–112)
Tail length	33	34.3±2.5 (25–36)	31.6±4.6 (25–35)
Spicules length	–	–	70.1±1.3 (68–72)
Ventromedian supplements	–	–	(13–17)

#### Description.

*Adult*. Slender nematodes of medium size, 1.92–2.40 mm long. Body cylindrical, slightly tapering towards both ends. Habitus more or less curved ventrad upon fixation, adopting an open ‘C’ shape. Cuticle dorylaimoid, with very fine transverse striation, sometimes difficult to distinguish; 3.5–5.0 μm thick in anterior region, 5.5–8.0 μm at mid–body and 8–13 μm on tail. Lateral chord 10–18 μm wide at mid–body, occupying 15–30% of mid–body diameter; lateral pores readily visible, arranged in two rows along both margins of lateral chord. Lip region truncate, somewhat angular, offset by marked constriction, 3.0–4.0 times as broad as high and 25–36% of body diameter at neck base; lips moderately separated; labial and cephalic papillae protruding. Amphid fovea funnel–like, its opening occupying 10–11 μm or three–fifths (60%) of lip region diameter. Odontostyle quite robust, wider than adjacent cuticle, 4–6 times as long as wide, 1.0–1.1 times the lip region diameter and 0.8–1.1% of body length; aperture occupying less than one–third (27–30%) its length. Guiding ring simple but distinct, at 10–12 μm or 0.5–0.7 times the lip region diameter from anterior end. Odontophore rod–like, lacking any differentiation, 1.1–1.4 times the odontostyle length. Pharynx consisting of a slender but muscular anterior portion enlarging gradually; basal expansion 9–11 times as long as broad, 5.3–6.0 times longer than body diameter at neck base, and occupying more than half (54–56%) of total neck length; pharyngeal gland nuclei situated as follows: DO = 46–50%, DN = 48–53%, S_1_N_1_ = 72–76%, S_1_N_2_ = 76–81%, S_2_N = 87–90%. Of note, S_1_N are significantly smaller than S_2_N. Nerve ring located at 155–187 μm or 32–41% of total neck length. Cardia tongue–like, 12–19 x 11–16 μm and surrounded by intestinal wall.

*Female*. Genital system didelphic-amphidelphic, with both branches well and equally developed, the anterior 364–476 μm long or 15–18% of total body length, and the posterior 358–395 μm long or 14–16% of total body length. Ovaries reflexed, moderately developed, sometimes reaching and surpassing the sphincter level; the anterior 114–131 μm, the posterior 101–127 μm long; oocytes arranged distally in several rows and then proximally in a single row. Oviduct joining ovary, 88–130 μm long or 1.3–1.8 body diameters and consisting of a tubular part and a moderately developed *pars dilatata* with small lumen. Oviduct-uterus junction marked by a distinct sphincter. Uterus 162–218 μm long or 2.3–3.5 times the corresponding body diameter, bipartite, *i.e*., consisting of two sections with variable length: a wider proximal region with distinct lumen containing abundant sperm cells, and a distal part with narrow lumen and globular walls surrounded by circular muscles. Vagina extending inwards 32–45 μm or less than half (36–44%) of the corresponding body diameter; *pars proximalis* longer than wide, 26–40 × 19–22 μm, with slightly sigmoid walls and surrounded by moderately developed musculature; *pars refringens* with (in lateral view) two distinct, trapezoidal, closely-spaced pieces, measuring 6–8 × 8–11 μm and with a combined width of 17–21 μm; *pars distalis* very short, with two small sclerotizations close to the *pars refringens*. Vulva a post–equatorial longitudinal slit appearing in lateral view as a short longitudinal depression. Prerectum 1.3–2.1 anal body diameters long, with a well developed blind sac. Rectum 0.9–1.3 times the anal body diameter. Tail short, conical with rounded tip to conoid, ventrally nearly straight, dorsally more convex. Two pairs of caudal pores, one subdorsal, other lateral or subventral.

**Figure 1.**
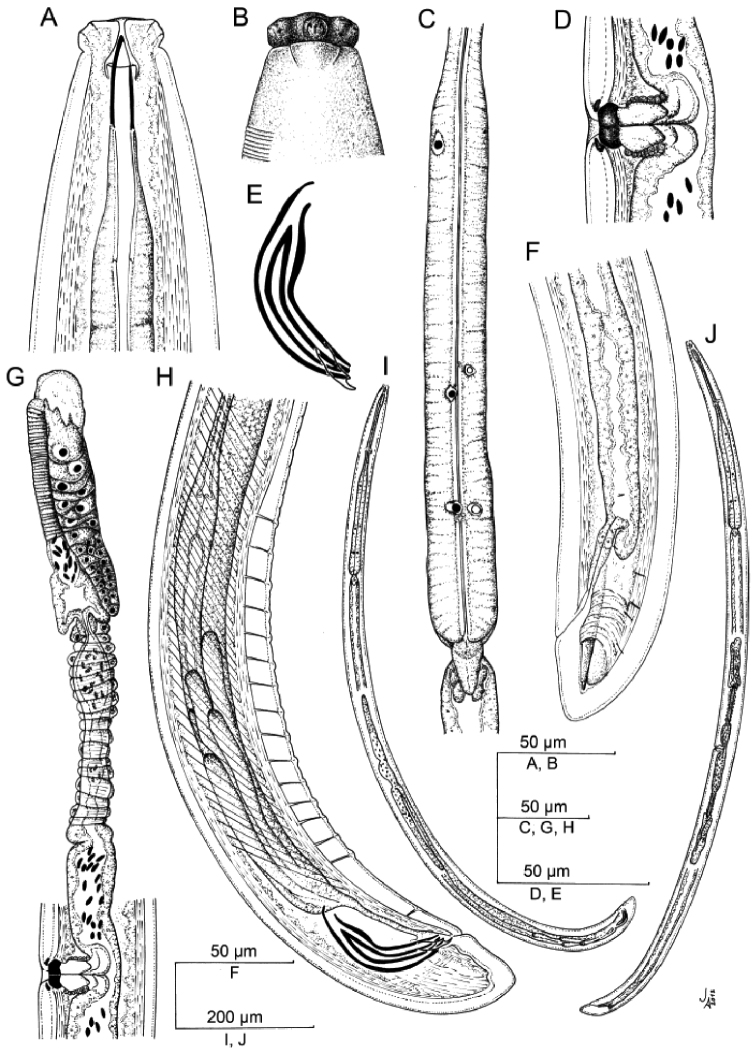
*Crassolabium persicum* sp. n. (all images are in lateral view) **A** Anterior region **B** Lip region and amphid fovea in surface **C** Pharyngeal expansion **D** Vagina **E** Spicules and lateral guiding piece **F** Female, posterior body region **G** Female, anterior genital branch **H** Male, posterior body region **I** Male, entire **J** Female, entire.

**Figure 2.**
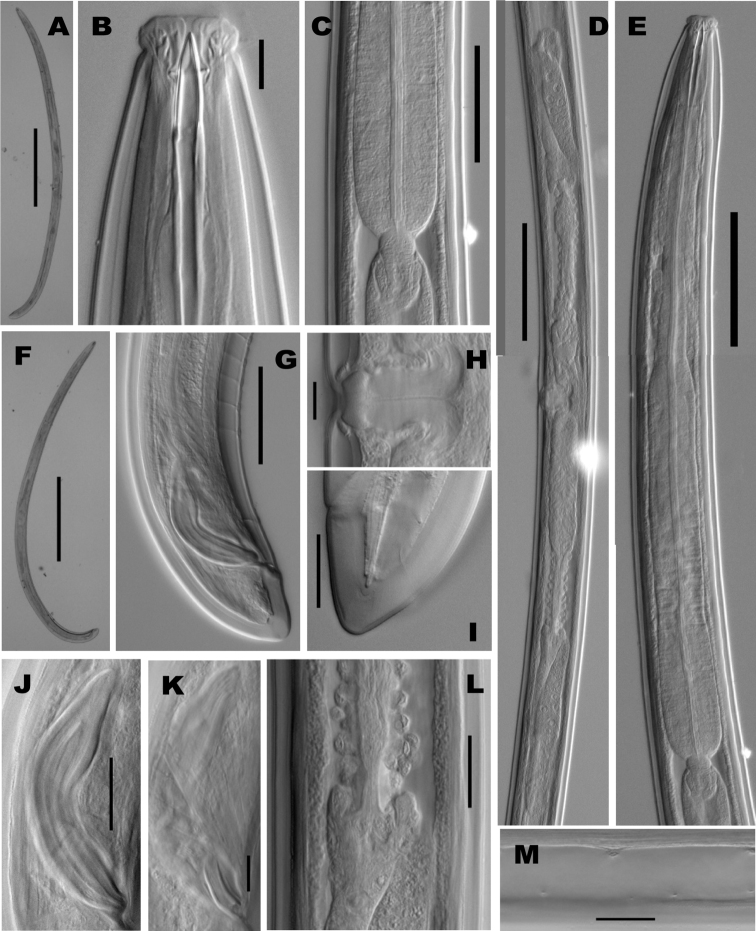
*Crassolabium persicum* sp. n. (light micrographs all in lateral view).**A** Female, entire **B** Anterior region **C** Pharyngo–intestinal junction **D** Female, genital system **E** Neck region **F** Male, entire **G** Male, posterior region **H** Vagina **I** Female, caudal region **J** Spicules **K** Lateral guiding piece **L** Oviduct–uterus junction **M** Lateral chord and pores. (Scale bars: **A, F** **–** 500 µm; **B, H, K –** 10 µm; **C, G** **–** 50 µm; **D, E** **–** 100 µm; **I, J, L, M** **–** 20 µm).

*Male*. Genital system diorchic, with opposite testes. In addition to the adcloacal pair, situated at 22–27 μm from cloacal aperture, there is a series of 13–17 shortly and regularly spaced (11–16 μm apart) ventromedian supplements, outside the range of spicules; posterior-most ventromedian supplement located at 47–53 μm from adcloacal pair. Spicules strongly curved ventrad and moderately robust, 4.0–5.0 times as long as wide and 1.6–1.7 anal body diameter long. Lateral guiding pieces short and robust, 11–16 μm long, 2.5–4.0 times as long as wide, with bifurcate terminus in which the posterior arm is visibly longer than the anterior one. Prerectum length 1.7–2.3 times the anal body diameter. Tail somewhat more conoid than that of female.

#### Diagnosis. 

The new species is characterized by body length of 1.92–2.40 mm , lip region offset by constriction and 17–19 μm wide, odontostyle 16–19 μm long with aperture occupying less than one-third (27–30%) its length, neck 428–690 μm long, pharyngeal expansion 369–390 μm long or occupying 54–56% of total neck length, female genital system amphidelphic, uterus bipartite and 162–218 μm long or 2.3–3.5 times as long as body diameter, *pars refringens vaginae* well developed, *V* = 54–57.5, vulva longitudinal, prerectum bearing a blind sac, tail conical with rounded tip to conoid (25–36 μm, *c* = 60–69, *c’* = 0.5–0.9), spicules 68–72 μm long, precloacal pair of supplements well far (22–27 μm) from cloacal aperture, and 13–17 shortly spaced ventromedian supplements with hiatus.

#### Relationships.

The general pattern of this species fits well with that of the genus *Crassolabium*. It resembles *Crassolabium diversum* (Ciobanu, Popovici, Abolafia & Peña-Santiago, 2007) Peña–Santiago and Ciobanu 2008, *Crassolabium major* (Thorne, 1974) Peña-Santiago and Ciobanu 2008, *Crassolabium montanum* (Ciobanu, Popovici, Abolafia & Peña-Santiago, 2007) Peña-Santiago and Ciobanu 2008 and *Crassolabium vietnamense* Vu, Ciobanu, Abolafia & Peña-Santiago, 2010. It differs from *Crassolabium diversum* in its larger body (*vs L* = 1.19–1.96 mm), longer pharyngeal expansion (*vs* 350–410 µm long or 40–48% of total neck length), vulva longitudinal (*vs* transverse), longer tail (*vs* 18–23 µm), longer spicules (*vs* 46–54 µm) and higher number of ventromedian supplements (*vs* 4–7). From *Crassolabium major* in its larger body (*vs L* = 1.5 mm), longer odontostyle (*vs* 13 µm long), vulva longitudinal (*vs* transverse), female tail more conical (*vs* rounded), longer spicules (*vs* about 50 µm) and higher number of ventromedian supplements (*vs* 9). From *Crassolabium montanum* in its larger body (*vs L =* 1.56–1.90 mm), lip region offset by constriction (*vs* continuous or offset by depression), shorter odontostyle (*vs* 20–22 µm), longer and bipartite uterus (*vs* 1.0–1.5 times the body diameter and simple), tail lacking (*vs* abundant) blister–like structures, and male as frequent as female (*vs* absent). From *Crassolabium vietnamense* in its larger body (*vs L =* 1.55–1.88 mm), shorter odontostyle (*vs* 19–22 µm), uterus bipartite (*vs* tripartite, with a distal, spherical region), vulva longitudinal (*vs* transverse), longer spicules (*vs* 53–55 µm), higher number of ventromedian supplements (*vs* 8–9), and hiatus present (*vs* two ventromedian supplements within the range of spicules).

The new species is also comparable to *Aporcelaimellus index* (Thorne, 1939) Andrássy 1986, in its eneral morphology and morphometry as well as the peculiar shape of lateral guiding pieces being short, robust and with bifurcate terminus in which the posterior arm is visibly longer than the anterior one, forming digitations, hence the specific name *index* as stated by Thorne (1939). *Aporcelaimellus index* is a very atypical member of *Aporcelaimellus* Heyns, 1965 because the odontostyle aperture is distinctly shorter than half its total length, making its identity questionable, and indicating further studies for its elucidation. It was originally described on the base of only one male specimen from Utah (U.S.A.), and later reported, also with only one male specimen, from Mongolia by Andrássy (1964). The new species herein described differs from *Aporcelaimellus index* in the position of the pre-cloacal pair of male genital papillae (*vs* closer to cloacal aperture) and in the number (*vs* 25–27) and arrangement (*vs* contiguous) of ventromedian supplements.

Finally, in the location of the pre-cloacal pair of male genital papillae - comparatively far from cloacal aperture, indeed a very unusual feature in dorylaims -the new species is reminiscent of the genus *Amblydorylaimus* Andrássy, 1998, with the type and only species *Amblydorylaimus isokaryon* (Loof, 1975) Andrássy 1998, but it differs from this in the nature of odontostyle (*vs* weakly sclerotized and longer, 31–35 µm long), S_1_N much smaller than DN and S_2_N (*vs* equally sized all of them), and hiatus present (*vs* absent).

#### Type habitat and locality.

The soil samples were collected from Mahmood Abad region (GPS coordinates: 38°48'N, 46°51'E), Arasbaran rangelands, East Azarbaijan province, north-west Iran.

#### Type material.

Female holotype, two female paratypes and three male paratypes are deposited in Nematode Collection, Faculty of Agriculture, University of Tabriz, Tabriz, Iran. One female paratype and one male paratype with nematode collection of Departamento de Biología Animal, Biología Vegetal y Ecología, Universidad de Jaén, Spain.

#### Etymology.

The specific epithet refers to the geographical area where the new species was collected from.

## Supplementary Material

XML Treatment for Crassolabium persicum
